# All-trans retinoic acid regulating angiopoietins-1 and alleviating extracellular matrix accumulation in interstitial fibrosis rats

**DOI:** 10.1080/0886022X.2021.1910046

**Published:** 2021-04-05

**Authors:** Zhiqing Zhong, Hong-Yan Li, Hongzhen Zhong, Wenshan Lin, Shujun Lin, Tianbiao Zhou

**Affiliations:** a Department of Nephrology, the Second Affiliated Hospital, Shantou University Medical College, Shantou, China; b Department of Nephrology, Huadu District People's Hospital of Guangzhou, Southern Medical University, Guangzhou, China

**Keywords:** All-trans retinoic acid (ATRA), renal interstitial fibrosis (RIF), angiopoietins-1 (Angpt-1)

## Abstract

All-trans retinoic acid (ATRA) is one of essentially active metabolite of vitamin A, and plays an important role in diverse physiological processes, such as cellular growth and function. Renal interstitial fibrosis (RIF) is a common pathological characteristic of chronic renal disease causing end-stage renal disease currently lacking effective treatment. Low level of Angiopoietins-1 (Angpt-1) is associated with extracellular matrix accumulation and fibrosis diseases. This study was performed to assess the association of ATRA with Angpt-1 in RIF disease. Rats were divided into three groups: group of sham (SHO group), group of unilateral ureteral obstruction group (UUO group), UUO mice administrated daily at the dose of ATRA (ATRA group). Masson-staining was used to detect the histologic lesion. Immunohistochemistry and Western-blot were applied to determine the targeted proteins. RIF score was significantly increased in UUO rats when compared with that of SHO group, and the fibrosis score was notably reduced in ATRA group. Transforming growth factor-β1 (TGF-β1), collagen IV (Col-IV) and fibronectin (FN) expressions in UUO group were significantly up-regulated, whereas Angpt-1 expression was significantly down-regulated compared with the SHO group. ATRA treatment reduced TGF-β1, Col-IV and FN expressions and improved Angpt-1 expression compared with the UUO group. The protein expression of Angpt-1 in kidney tissue of UUO group was negatively correlated with RIF index and protein expressions of Col-IV, FN and TGF-β1. In conclusion, low expression of Angpt-1 was associated with the RIF disease and ATRA treatment can increase the Angpt-1 and alleviate the RIF lesion in UUO rats.

## Introduction

All-trans retinoic acid (ATRA) is one of essentially active metabolite of vitamin A, and plays an important role in diverse physiological processes, such as cellular growth and function [1–3]. Vitamin A deficiency can lead to extracellular matrix (ECM) and over-expression of collagen IV (Col-IV) and fibronectin (FN) [4]. ATRA acts as a negative regulator of activation of renin-angiotensin-aldosterone system and alleviates the accumulation of ECM accumulation to prevent renal interstitial fibrosis (RIF) [5]. ATRA can downregulate the myofibroblast differentiation, ECM accumulation, cell migration, and alpha-smooth muscle actin (α-SMA), collagen type 1, FN in TGF-β1-induced nasal polyp-derived fibroblasts [6].

RIF is a common pathological characteristic of chronic renal disease causing end-stage renal disease currently lacking effective treatment, which is also characterized by an imbalance between the synthesis and the degradation of the collagen-rich ECM and massive ECM deposition caused by activated fibroblasts and myofibroblasts [7–10]. The global prevalence of chronic kidney disease (CKD) at 13.4%, and the awareness of CKD remains is only 6% in the general populations [11]. There still is no effective drug and therapy to prevent and treat the patients with renal fibrosis diseases in CKD patient. Thus, to address this problem, there is an urgent need to identify some possible biomarkers and explore its mechanisms. Furthermore, whether there is a new drug to treat it is also needed to detect. In our previous study in RIF rats induced by unilateral ureteral obstruction (UUO), we found that ATRA alleviated the RIF lesion but not by the signaling pathway of transforming growth factor-β1 (TGF-β1)/Smad3 [12]. It indicated that the action mechanism of ATRA is complicated and there might be other signal pathway for ATRA on RIF rats induced by UUO.

Angiopoietins-1 (Angpt-1) is a major pro-angiogenic factor [13], and is involved in angiogenesis and vascular remodeling by establishing vascular integrity [14–16]. Low level of Angpt-1 is associated with ECM accumulation and fibrosis diseases [17,18]. However, there was no study to assess the relationship between ATRA and Angpt-1 in UUO model. In this context, this study was conducted to assess whether ATRA can regulate Angpt-1 to alleviate the RIF lesion. Herein, we investigated the potential association between ATRA treatment and Angpt-1 in RIF disease induced by UUO in rats, and assess whether Angpt-1 was associated with the expression of transforming growth factor-β1 (TGF-β1), Col-IV and FN.

## Materials and methods

### Experimental design

Thirty male rats (8 weeks old, 172 ± 16 g) were randomly divided into 3 groups: A) sham (SHO) group; B) unilateral ureteral obstruction (UUO) group; C) ATRA treat (ATRA) group, 10 rats per group. Before surgery the rats were performed under general anesthesia (40 mg/kg ip sodium pentobarbital). The left ureter was either completely ligated with two separate silk ties without cutting off the ureter (group B/C) or manipulated similarly but not ligated (group A). ATRA dissolved in corn oil at a dose of 15 mg/kg (group C) or vehicle corn oil (group A/B) was orally administered from 3 days before surgery and on each day until rats were sacrificed after 14 and 28 days (*n* = 5 for each time point for all groups). Kidneys were removed for histological analysis or Western blot assay.

### Reagents

Anti-Angpt-1 antibody was purchased from Abcam, anti-TGF-β1, anti-FN, anti-Col-IV antibody and ECL were obtained from Santa Cruz Biotechnology. BCA protein assay kits and RIPA lysis buffer were purchased from Beyotime.

### Histologic examination

After removed kidneys, we fixed and embedded them with 10% neutral formalin and paraffin, respectively. Then we sectioned the tissue into 4.0 μm thickness, and stained it with H&E and Masson trichrome. Under × 400 magnification, we analyzed tubulointerstitial fibrosis through 20 selected fields randomly from each section. We scored their degree of tubulointerstitial fibrosis according to the ratio of the positive stain area (blue) of the whole vision (*n* = 20). RIF index was obtained by the method described in our previous study [12]. Besides, we examined tubulointerstitial fibrosis of every rat and averaged for the five rats of each group.

### Immunohistochemistry

The protein expressions of Angpt-1, TGF-β1, Col-IV and FN were determined by immunohistochemistry method. After incubating kidneys in citrate buffer for 3 min at 100 °C, they were then sectioned into 4.0 μm thickness. At room temperature, we rinsed sections in PBS and blocked endogenous peroxidase with 3% H_2_O_2_ for 30 min. To exclude the interference of the nonspecific antibodies, we incubated them with 1% pre-immune serum in PBS for 1 h at 37 °C. Then we immunostained sections with antibodies at 4 °C overnight. We rinsed the sections in PBS and incubated with horse radish peroxidase (HRP) conjugated secondary antibody for 1 h at ambient temperatures. Next, at ambient temperatures, bound antibodies were exposed with diaminobenzidine/H_2_O_2_ staining for 3 min. Finally, we countered stained slides with hematoxylin, and captured digital images with a light microscope instrumented with a camera (ZEISS Imager A2). The immunohistochemical results were analyzed and numerical data were obtained by the method described in our previous study [12].

### Western-blot

Angpt-1 protein was detected used western-blot method. Mixed minced kidney tissues and RIPA lysis buffer thoroughly for 30 min at low temperature, and then centrifuged at 12000 rpm at 4 °C for 15 min. Precipitate was removed and the supernatant was collected. A standard curve was developed with a BCA Protein Assay Kit to determine the protein concentration of samples. Dilute 5× SDS-PAGE sample loading buffer to 1× with the whole lysates and boiled them for 5 min to denature protein. An equal amount of protein was sampled into the SDS-polyacrylamide gels. Proteins with different molecular weights were separated by electrophoresis and then transferred to a PVDF membrane. Incubated the membrane blots with primary antibody and horseradish peroxidase-conjugated second antibody in sequence. The enhanced chemiluminescent system was used to visualize the protein antigen for quantitative data. The comparable definition was based on the signal intensity of β-actin, an internal control. The density of the western-blot bands was analyzed and numerical data were obtained by the method described in our previous study [12].

### Statistical analysis

All data (mean ± SD) were analyzed by SPSS 11.5 software. Statistical significance was evaluated by one-way ANOVA with *post hoc* contrasts by Student–Newman–Keuls test and Pearson linear correlation analysis. *p* < 0.05 were considered as statistical significance.

## Results

### ARTA improves histopathologic changes in UUO rats

**Figure 1.**
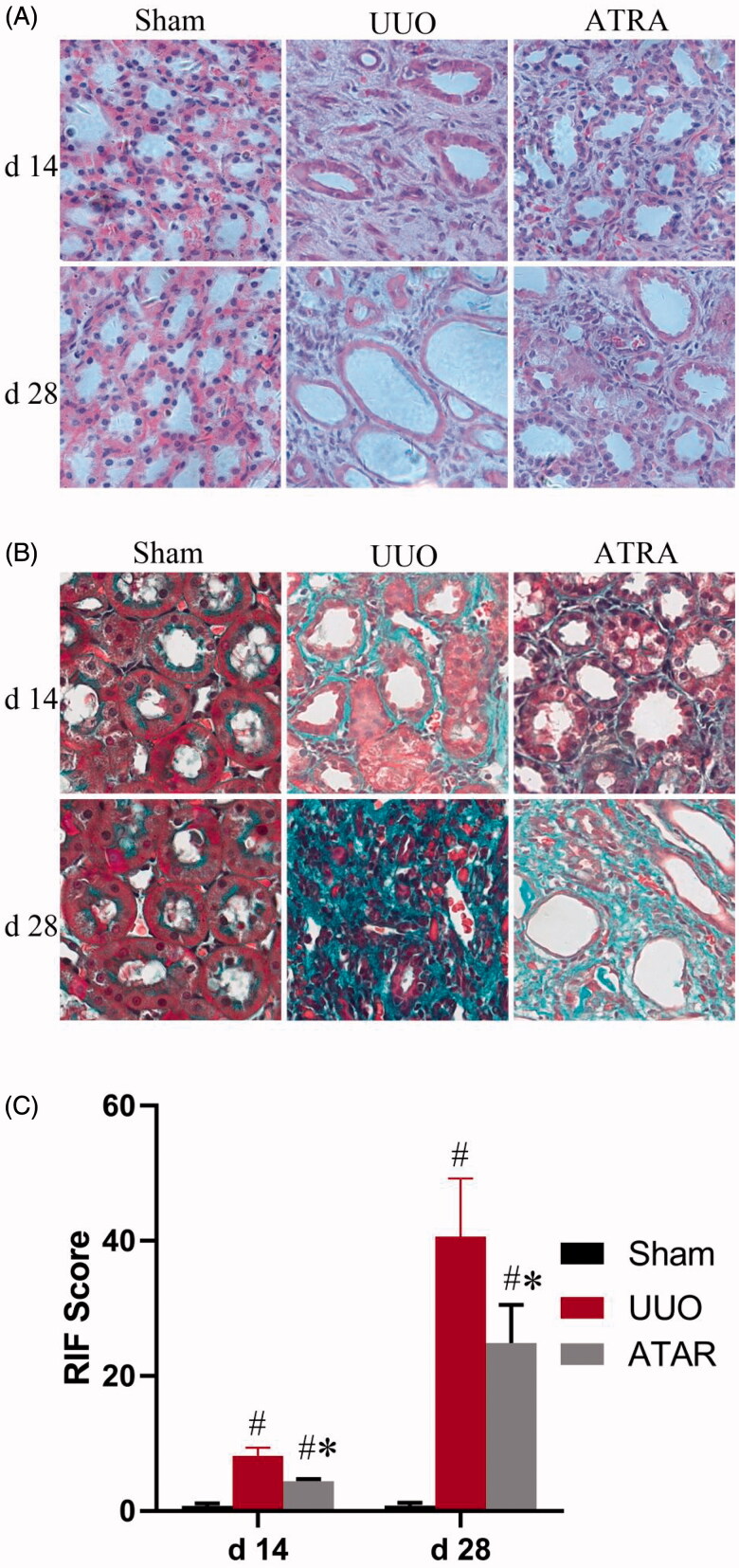
Representative histopathology in sham rats, UUO rats and ATRA rats at d 14 and d 28, respectively. A Hematoxylin-eosin staining showing abscission of the epithelial cells, tubular ectasia, and inflammatory cells infiltrating in interstitial space (Hematoxylin-eosin stain, ×400). B Masson staining showing moderate and severe fibrosis after ligation (Masson stain, ×400). **C** RIF score was markedly increased in UUO rats and reduced in ATRA rats at d 14 and d 28. ^#^*p* < 0.05 vs sham group; *p < 0.05 vs UUO group.

After ureteral ligation, the major signs of renal damage were hydronephrosis and thin renal cortex. From d 14 to d 28, the renal tubular dilation, loss of integrality for tubular brush border, and severity for inflammatory infiltration was significantly enhanced in UUO group when compared with SHO group (Figure 1(A)). In parallel, the renal interstitial area gradually widened and fibrosis gradually developed in UUO group, and the ATRA group was weaker than UUO group, as showed by the staining of Masson trichrome (Figure 1(B)). Meanwhile, RIF score was notably up-regulated in UUO rats when compared with that of sham rats. Compared with UUO rats, the RIF score was markedly down-regulated in rats of ATRA group at d 14 and d 28. The values were shown in Figure 1(C).

### ATRA affects the expressions of angpt-1, TGF-β1, Col-IV and FN in UUO rats

**Figure 2.**
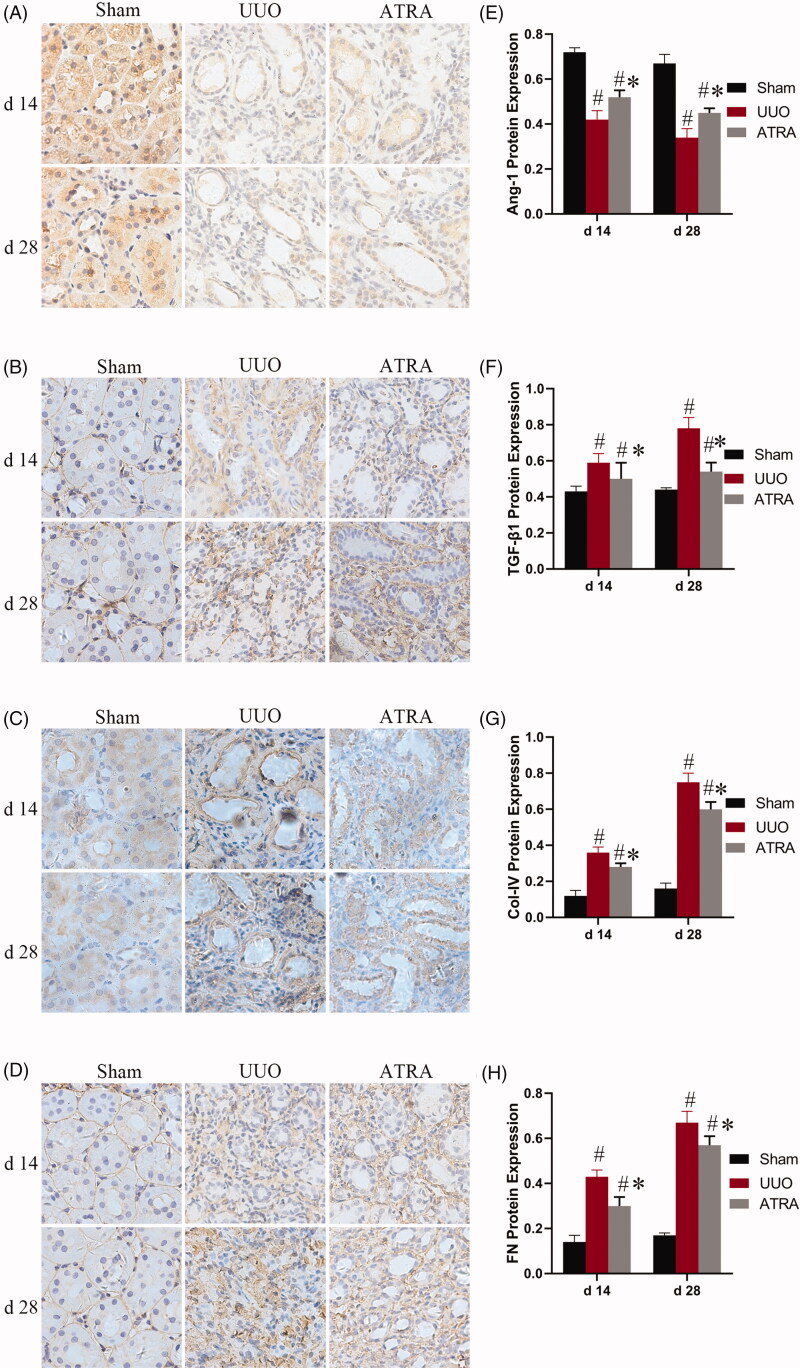
The expressions of Angpt-1, TGF-β1, Col-IV and FN protein in sham rats, UUO rats and ATRA-treated rats at d 14 and 28, respectively. (A, E) Representative immunohistochemistry of Angpt-1 in the kidney. (B, F) Representative immunohistochemistry of TGF-β1 in the kidney. (C, G) Representative immunohistochemistry of Col-IV in the kidney. (D, H) Representative immunohistochemistry of FN in the kidney. Results were shown as average optical density for protein and presented as mean ± SD. #*p* < 0.05 vs sham group; *p < 0.05 vs UUO group.

An imbalance of ECM synthesis and degradation was an important event in RIF, including expression of cytokines (e.g., TGF-β1) [19,20] and mesenchymal markers (e.g., Col-IV and FN) [21,22]. To assess the effect of ATRA on ECM in the renal interstitial, Angpt-1, TGF-β1, Col-IV and FN protein expression levels were determined by immunohistochemistry in UUO rats treated with or without ATRA, respectively. As shown in Figure 2, in the group of UUO, TGF-β1, Col-IV and FN expressions were significantly up-regulated, whereas Angpt-1 expression was notably down-regulated than the group of SHO from d 14 to d 28 (*p* < 0.05). ATRA treatment can reduce TGF-β1, Col-IV and FN expressions and improved Angpt-1 expression at d 14 and d 28 than the UUO group (*p* < 0.05; Figure 2).

### ATRA restores angpt-1 expression in UUO rats

**Figure 3.**
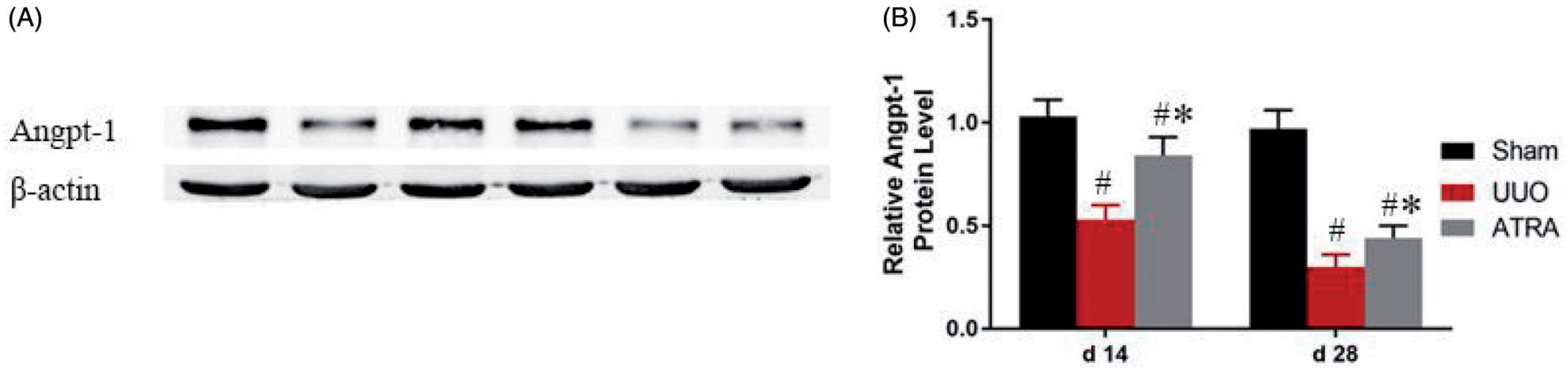
The expressions of Angpt-1 protein in sham rats, UUO rats and ATRA-treated rats at d 14 and d 28, respectively. (A) Representative Western blot of Angpt-1 in sham and UUO rats treated with or without ATRA. (B) Compared with UUO group, ATRA treatment significantly increased Angpt-1 expression. Results were shown as ratio of optical density for Angpt-1 to that of β-actin and presented as mean ± SD. #*p* < 0.05 vs sham group; *p < 0.05 vs UUO group.

To investigate the effect of ATRA on ECM accumulation in the RIF rats, Angpt-1 protein expression was determined using Western-blot method. As shown in Figure 3, when compared with the group of sham, the expression of Angpt-1 in UUO group was markedly down-regulated from day of d 14 to d 28 (*p* < 0.05; Figure 3). Interestingly, ATRA treatment in group of UUO resulted in increased the expression of Angpt-1 on days 14 and 28 compared to the group of UUO (*p* < 0.05; Figure 3).

### Correlation analysis

The protein expression of Angpt-1 in kidney tissue of UUO group was negatively correlated with RIF index and protein expressions of Col-IV, FN and TGF-β1 (*r* = −0.758, −0.741, −0.725, −0.680; all *p* < 0.01).

## Discussion

In this study, the RIF disease model was conducted successfully. The ECM accumulation and the increased expressions of TGF-β1, Col-IV and FN were observed in the UUO group. The renal fibrosis was happened in the UUO rats. We used this model to assess the role of Angpt-1 in the pathogenesis of RIF disease. We found that the low express of Angpt-I was associated with the ECM accumulation and Angpt-I might be a protective factor against RIF lesion.

Angpt-1 is a secreted vascular growth factor, and its low expression is associated with ECM accumulation and fibrosis diseases. Singh et al. [23] conducted a study in a murine UUO model and reported that enhanced renal tubular Angpt-1 expression can halt the progression of renal fibrosis which was association with the decreased renal inflammation and upregulated peritubular capillaries. Loganathan et al. [24] reported that Angpt-1 deficiency increased renal tubulointerstitial fibrosis in mice, and they found that the expression of α-SMA, TGF-β1, collagen type 1 α1 and FN in Angpt-1 deficient kidneys had significantly more compared to wild type mice after UUO. These studies indicated that the Angpt-1 might be a protective factor against the renal fibrosis. In our study, we found that the expression of Angpt-1 was reduced in the RIF rats when compared with normal control rats. Furthermore, the protein expression of Angpt-1 in kidney tissue of UUO group was negatively correlated with RIF index and protein expressions of Col-IV, FN and TGF-β1. These results were similar when compared with those from these studies mentioned above.

ATRA is reported to be an anti-fibrosis agent in many diseases. In past decades, studies indicated that the ATRA can alleviate the fibrosis in pulmonary fibrosis [25], diabetic nephropathy [1,26], etc. In our studies, we found that ATRA can reduce the RIF index and down-regulate the protein expressions of Col-IV, FN and TGF-β1. Furthermore, ATRA can alleviate the fibrosis of UUO rats. Interestingly, we also found that ATRA can increase the expression of Angpt-1 in UUO rats and alleviate the fibrosis. Li et al. [27] found that ATRA treatment can reduce the Angpt-1 expression in tissue of xenograft tumors from mice, indicating the mechanism of action for ATRA treatment in cancer angiogenesis *in vivo*. However, there was no report focused on the association of ATRA treatment with Angpt-1 in renal fibrosis diseases. Our study might be the first investigation focusing on this topic.

However, there were limitations in this study. There was no normal control group in our study. The cell culture to explore the effect of ATRA on Angpt-1 *in vitro* has not been carried out in this study. More studies should be conducted in the future to confirm the effect of ATRA on Angpt-1 in RIF disease.

To sum up, in this study, low expression of Angpt-1 was associated with the RIF disease and ATRA treatment can increase the Angpt-1 and alleviate the RIF lesion in UUO rats.
